# Fact-checks focus on famous politicians, not partisans

**DOI:** 10.1093/pnasnexus/pgae567

**Published:** 2024-12-19

**Authors:** Kevin T Greene, Nilima Pisharody, Faelynn Carroll, Jacob N Shapiro

**Affiliations:** Empirical Studies of Conflict, Princeton University, Princeton, NJ 08544, USA; Department of Economics, New York University, New York, NY 10003, USA; Empirical Studies of Conflict, Princeton University, Princeton, NJ 08544, USA; Empirical Studies of Conflict, Princeton University, Princeton, NJ 08544, USA

**Keywords:** fact-checking, social media, partisanship

## Abstract

Does the fact-checking enterprise focus its attention on one party? If Republican or Democratic politicians were systematically more likely to have their statements evaluated, that would call into question both the impartiality of the fact-checking enterprise and the results of the many papers that rely on fact-checks to drive other measurements. Despite frequent claims that fact-checking organizations are biased against Republicans, there is little systematic evidence regarding political bias in this industry. We address these gaps using data on how often each member of Congress was fact-checked from 2018 to 2021. We construct measures to account for multiple factors theorized to influence fact-checking, including a member’s partisanship, prominence, and the quality of the news sites they link to. We find that Republican elected officials are not fact-checked more often than Democratic officials. Politician prominence predicts fact-checking, but partisanship does not. Our findings suggest it is unlikely that the selection approach used by fact-checking groups creates partisan bias in fact-check-derived measures.

**Significance Statement**

While fact-checks are viewed as a key component of a healthy information environment, there are frequent claims that fact-checking organizations more heavily scrutinize Republicans relative to Democrats. This risks undermining trust in the process and introduces biases into measures derived from fact-checks. Across assessments, we find little evidence that Republican members of Congress are fact-checked at a higher rate than Democrats. Instead, fact-checks overwhelming focus on highly prominent members (political leaders and those more frequently covered in the media), narrowing the information available to support democratic accountability by the electorate.

## Introduction

Fact-checks play an important role in the overall health of the information environment, by helping online audiences navigate historic volumes of information, both true and false (1–4), and improving the accuracy of individuals’ factual beliefs (5, 6). Further, fact-checks help bolster democratic accountability (7, 8), by providing clarity to the electorate about the claims made by political leaders (9). The work of fact-checking groups is increasingly used in academic research to study the quality of online information (1, 4) and to train models for automated fact-checking (10–16).

Although research has found no biases in the selection of news stories covered (17), there have been frequent allegations that political biases within fact-checking organizations in the United States lead them to be more likely to scrutinize the statements of Republicans relative to Democrats (18–22). Claims range from fact-checkers refusing to cover inconvenient truths (20) to serving as “propagandists” (23). However, leaders of fact-checking organizations dispute these claims (24, 25). PolitiFact founder Bill Adair, for example, notes that news judgment drives the decisions of the organization about which statements to check, and they aim to balance checks across political parties (26).

Systematic political biases in which claims are fact-checked would pose fundamental problems for advancing factual knowledge and scientific research. In particular, measures constructed from fact-checking data would be differentially accurate for statements from one party, calling into question a wide range of work that builds on fact-checking organizations’ analyses (10, 12, 13, 16, 27–30), while risking encoding selection biases in automated fact-checking systems (10, 12, 13, 16, 27), potentially powered by black box AI (31–33). Moreover, these biases may undermine the effectiveness of fact-checking groups. Past work finds that conservatives are the least trusting of fact-checking (5, 30, 34), yet these individuals consume and share most of the unreliable news sites (35–37). Thus, the individuals who might receive the most novel information from fact-checks are the least trusting of the process. Finally, biases that draw attention to particular officials necessarily mean that others will be checked at a lower rate. This creates inequality in the information available for the electorate to hold representatives accountable.

However, a lack of statistically grounded research leaves two substantial gaps in our understanding of possible biases in the fact-checking enterprise. First, previous work studying potential bias has relied on small or unrepresentative samples of fact-checks (38–41). Many of the past claims about biases are supported by evaluating the fact-checks of a few prominent political officials such as Presidents Trump, Biden, or Obama (18, 19, 22, 29). Further, this work often selects on the dependent variable, by only evaluating officials that have received fact-checks (20, 21, 38, 41). Without a systematic sample selection strategy, we do not know if these findings support broader claims about biases in fact-checking.

Second, past work has failed to account for additional individual or organizational factors that might influence fact-checking of elected officials (20, 21). Media reports alleging bias in fact-checking often implicitly assume that elected officials differ on only a single salient dimension: party identification. However, if differences exist in the behavior of elected officials, or in the constraints faced by fact-checking groups, then current conclusions could be spurious.

We fill these gaps first by identifying factors that have been suggested to influence fact-checking. Partisanship has been noted as a key driver of fact-checking scrutiny (1, 42), especially in its most extreme form (43–45). As detailed previously, there are frequent claims that Republicans are more heavily scrutinized by fact-checking groups relative to Democrats (18–22).

Political leaders have also been found to receive increased scrutiny (4, 8, 29, 38, 39, 46). Further, the “gatekeeping” literature has consistently found that political leaders receive more attention than nonleaders (43–45, 47).

Coverage in news media is also likely to influence the rate of fact-checking. First, fact-checking is an off-shot of journalism (48) and arose partially in response to increasing rates of dubious information in politics (26, 49–51). Second, media coverage of statements can make fact-checking more likely both by adding statements to the public record and by increasing their coverage, thus increasing the impact of a possible fact-check (26, 43–45, 47, 48).

The social media activity of members of Congress is also likely to influence the rate of fact-checking. Currently, most members of Congress actively use social media (52), and these platforms are used by members of Congress to engage with journalists and constituents (53).

Finally, as members may receive more fact-checks simply because they publicize more questionable material (4, 40, 54), we also account for the quality of the content shared.

And then, we analyze whether politicians’ partisan identity impacts the rate at which they are fact-checked after accounting for a range of potential confounders. We focus on partisanship as it could contaminate measures of members’ overall truthfulness and impact the generalizability of measures derived from fact-checks. To do so, we first collect data on how often every US member of Congress from 2018 to 2021 was fact-checked by PolitiFact, the largest fact-checking organization. We use these data to construct yearly measures of the number of fact-checks of each official. We construct a series of measures to account for multiple factors that have been suggested to influence fact-checking, namely a member’s partisanship, prominence (political leaders and those more frequently covered in the media), and content quality.

In contrast to many expectations, we find no evidence that Republican elected officials are fact-checked more often than Democratic officials. This relationship is consistent across numerous model specifications and functional forms. Instead, we find that more prominent members of Congress receive the overwhelming majority of fact-checks, regardless of party. The biases in fact-checking appear to revolve around the popularity of the member, not their party identification.

Overall, fact-checking of members of Congress is highly unequal. Most representatives in our study received zero fact-checks, and 20% of representatives received fully 90% of total fact-checks. Vermont Senator Bernie Sanders received more fact-checks than the combined members of Congress from 22 states. Politicians representing most of the United States (particularly the geographic middle of the country) received few fact-checks.

## Results

**Fig. 1.**
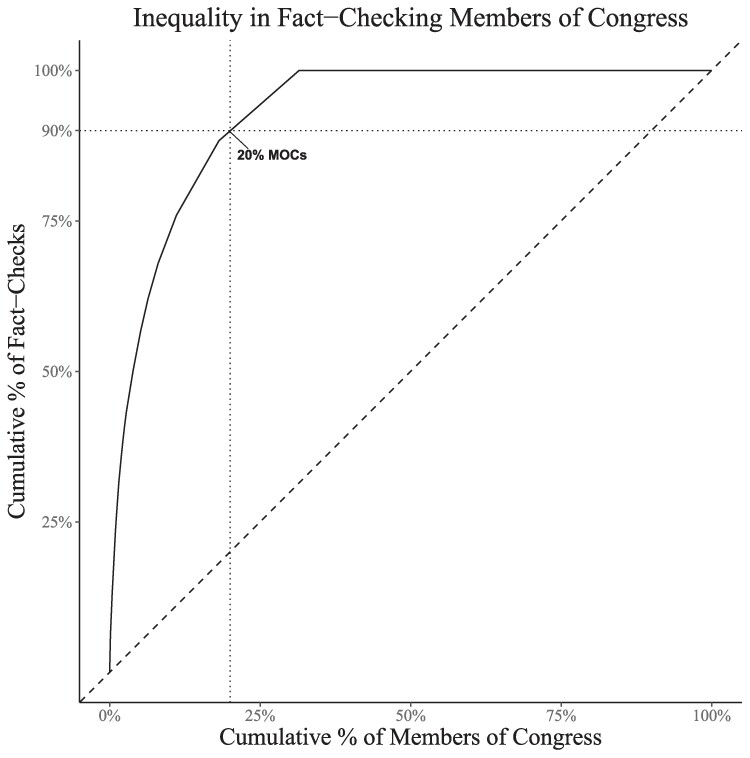
Concentration of fact-checks among members of Congress. A curve laying on the 45° line would indicate equality in fact-checking (i.e. 10% of the members receiving 10% of total fact-checks), while a curve above the 45° line indicates that fact-checks are concentrated among a smaller subset. The intercept of the two dotted lines shows that 20% of members of Congress receive 90% of total fact-checks. Fact-checking data are from PolitiFact and aggregated to the member level.

Fact-checking members of Congress are highly concentrated on a relatively small number of officials (Fig. 1). Only 20% of members account for 90% of the total fact-checks, and most members of Congress receive no fact-checks. As presented in Fig. 1, a relatively small number of members appear to be fact-checked at a higher rate, regardless of political party.

**Fig. 2.**
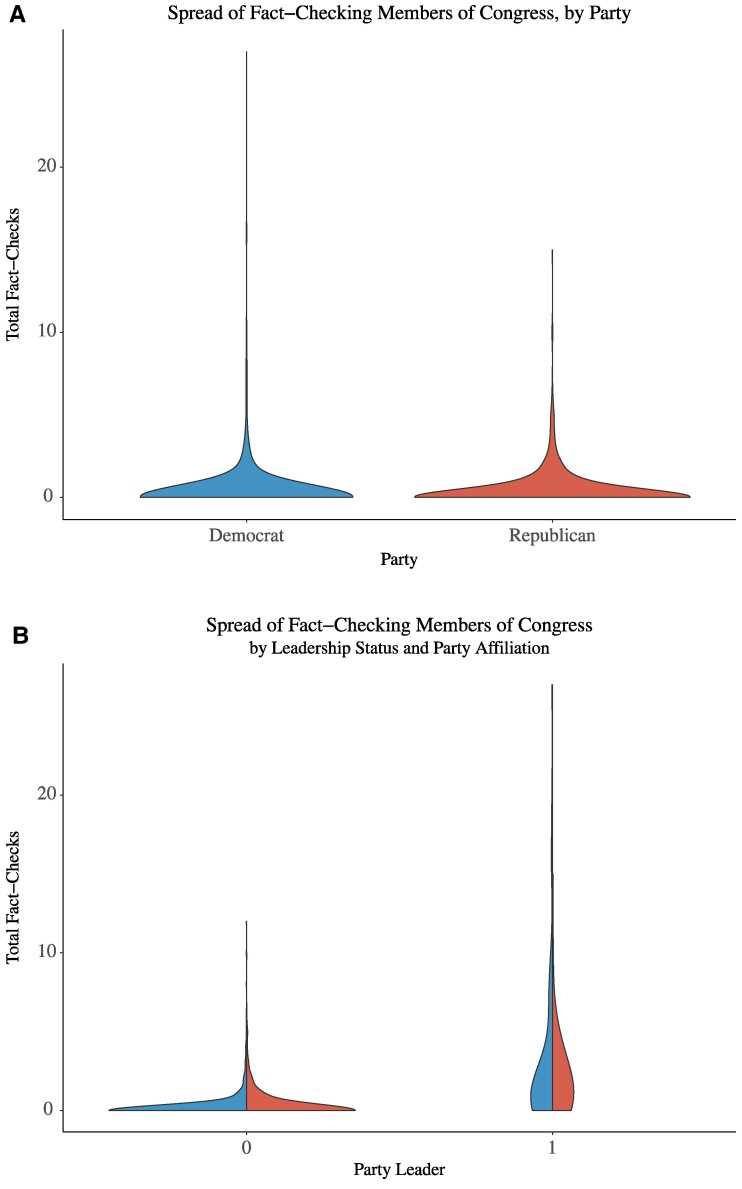
The distribution of fact-checks of members of Congress across party and leadership. A) The distribution of the number of fact-checks for Republican and Democratic members of Congress. B) The distribution of the number of fact-checks for political leaders and nonleaders. The unit of analysis is the member-year. Fact-check data are from PolitiFact.

We find little evidence that Republican officials are fact-checked more often than Democratic officials (Fig. 2A). In fact, the members receiving the largest number of fact-checks are Democrats. However, when we break down these results to account for a member’s leadership status, we see leaders are fact-checked far more than nonleaders (Fig. 2B). In the Supplementary material, we conduct analyses with different definitions of party leadership and find consistent results.

**Fig. 3.**
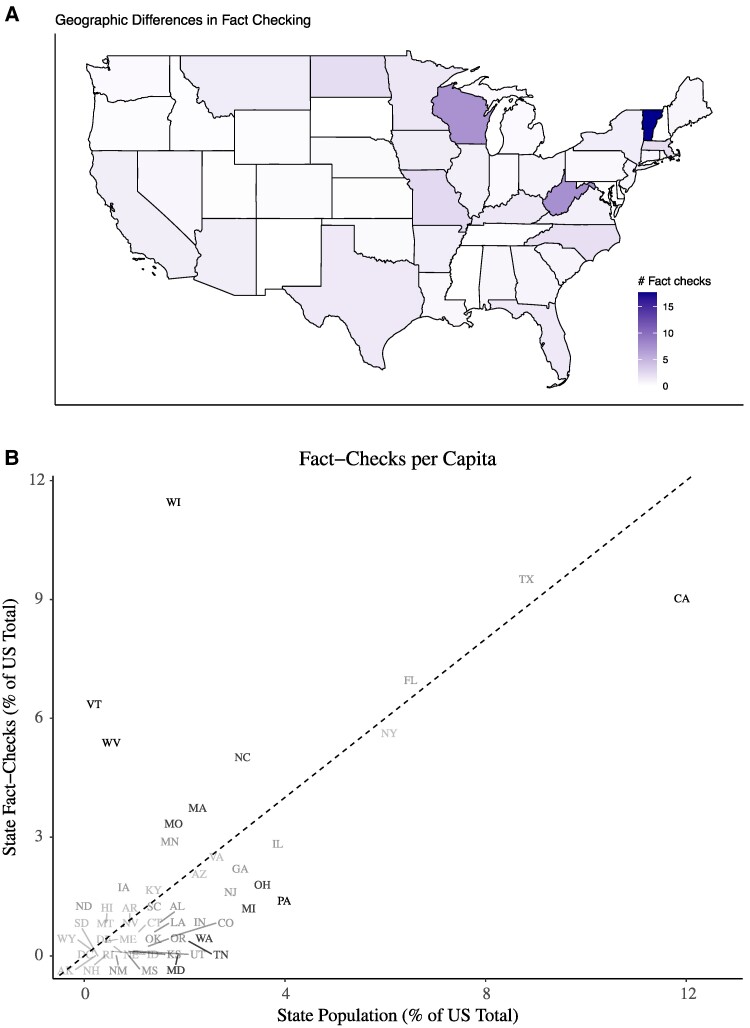
The geographic distribution of fact-checks of members of Congress. A) The number of fact-checks per member year for each US state. Locations where members are checked more often are darker. B) The 45° line shows states where fact-checks per capita are equal to the state’s percentage of the US total population. States with darker labels indicate large deviations from this value. States above or below the line are overrepresented or underrepresented, respectively. Fact-check data are from PolitiFact. Population data are sourced from the 2020 US Census.

We further examine the geographic representation of fact-checking first in Fig. 3B which measures fact-checks per capita across the United States. Comparing the allocation of fact-checks to the allocation of the population across the United States reveals that the majority of states’ members of Congress received less than one fact-check per member from 2018 to 2021. Members of Congress from Arkansas, Nebraska, South Dakota, and Wyoming received no fact-checks over this period. The states of Maryland, Tennessee, Washington, Michigan, Ohio, and Pennsylvania (bottom left of Fig. 3B) are particularly underrepresented in their rates of fact-checking compared with their population share. The few states that lie along the diagonal line shaded in gray, New York, Florida, and Texas, all have large population shares and receive similarly high rates of fact-checks. Above the diagonal line, we find notably overrepresented states with relatively lower populations and higher fact-checking rates, which include West Virginia, Wisconsin, and Vermont. Despite these three states accounting for 2.51% of the US population, members from these states account for 23.45% of the total fact-checks.

Similar patterns emerge when investigating the spatial distribution of fact-checks (Fig. 3A). Here states with fewer fact-checks per member are indicated by lighter shades of purple, and states with more fact-checks per member are indicated by darker shades. PolitiFact’s fact-checking attention being focused on a few states leaves the majority of the country largely unchecked.

To further evaluate these relationships, we use linear regression to measure the association between the yearly number of fact-checks received by each member of Congress and a binary indicator for whether a member is a Republican. We include measures for other factors that could influence the rate of fact-checks including party leadership, member’s media prominence, the quality of their online content (toxicity of posts and proportion of links to low-quality news sources), their social media presence (the log number of Facebook followers and the log number of Facebook posts), and their tenure in Congress. Descriptive statistics for the variables included in our models can be found in Table 1.

**Table 1.** Descriptive statistics.

	Mean	SD	Min	Max	*N*
**Dependent variable**					
Fact-checks per year	0.379	1.393	0.000	27.000	2,170
**Partisanship**					
Republican	0.496	0.500	0.000	1.000	2,170
Partisanship	0.434	0.152	0.000	0.936	2,170
**Media prominence**					
News mentions (log)	1.999	1.399	0.000	6.483	2,170
**Political leadership**					
Leader	0.047	0.212	0.000	1.000	2,170
**Social media**					
FB followers (log)	8.918	3.459	0.000	16.013	2,170
FB posts (log)	4.226	1.784	0.000	7.473	2,170
**Content quality**					
Low-quality link share	0.011	0.036	0.000	0.556	2,170
Toxicity	0.037	0.024	0.000	0.213	2,170
**Structural controls**					
State fact-check	0.502	0.500	0.000	1.000	2,170
Tenure	10.681	9.123	1.000	49.000	2,170

The unit of analysis is a member of Congress-year. Variables are scaled for comparability. Missing values are imputed as zero. The unit of observation is a member of Congress-year. Errors are clustered by member. Dynamic weighted (DW)-Nominate (partisanship) scores are sourced from Voteview. Fact-checks are from PolitiFact. Facebook posts were processed with Perspective application programming interface (API) to generate measures of toxicity. News source quality ratings are from Media Bias Fact Check. News mentions counts mentions of each member of Congress from AP News Wire.

**Fig. 4.**
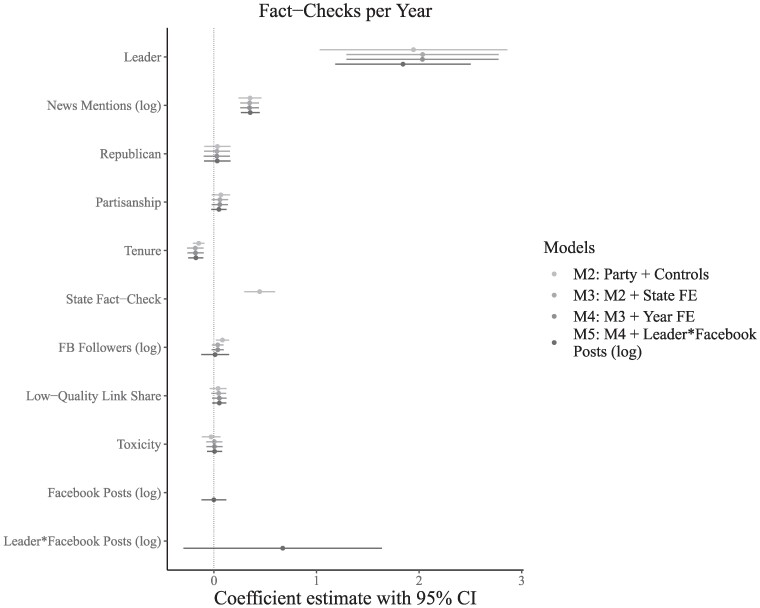
Regression coefficients and 95% CI for correlates of the number of fact-checks. Standard errors are clustered by member of Congress. Variables are scaled for comparability. Missing values are imputed as zero. The unit of analysis is a member of Congress-year. Errors are clustered by member. DW-Nominate (partisanship) scores are sourced from Voteview. Fact-checks are from PolitiFact. Facebook posts were processed with Perspective API to generate measures of toxicity. News source quality ratings are from Media Bias Fact Check. News mentions counts mentions of each member of Congress from AP News Wire.

We find no evidence that Republicans are fact-checked at a higher rate than Democrats, as shown in Fig. 4. Within the plot, M2 includes the variables mentioned above. M3 adds state fixed effects to M2 to account for unobserved between-unit heterogeneity. M4 adds year fixed effects to M3 to account for trends over time. Finally, M5 adds an interaction between the number of posts shared by a member of Congress and their leadership status. Across models, standard errors are clustered on the member of Congress.

The coefficient indicating members of the Republican party is not statistically significant at conventional levels when accounting for potential confounders (M2: β=0.033, 95% CI, −0.094 to 0.160), after the inclusion of state fixed effects (M3: β=0.029, 95% CI, −0.096 to 0.154), after including state and year fixed effects (M4: β=0.028, 95% CI, −0.099 to 0.155), or after accounting for the total posts shared by members of Congress (M5: β=0.032, 95% CI, −0.097 to 0.161).

Being among the leadership in Congress is consistently associated with being fact-checked at a higher rate. Leaders receive roughly two additional fact-checks per year (M2: β=1.945, 95% CI, 1.030 to 2.860), while controlling for factors including partisanship, social media presence and content quality, structural variables, and state and year fixed effects. Further, a member’s coverage in the media is consistently associated with being fact-checked at a higher rate (M2: β=0.352, 95% CI, 0.240 to 0.464), while controlling for factors including partisanship, social media presence and content quality, structural variables, and state and year fixed effects. These findings are consistent when using an alternative definition of political leaders, when using Poisson regression, after accounting for outliers by log transforming or winsorizing the outcome variable, after accounting for differences in the content shared across parties, and after accounting for the gender of the member of Congress. These results are presented in the Supplementary material.

**Fig. 5.**
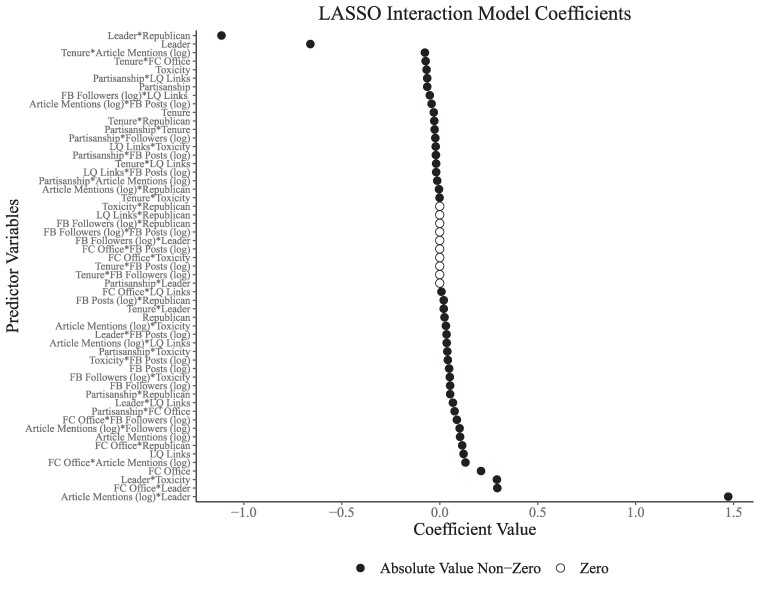
LASSO coefficients for pair-wise interactions of model features. Features are taken from M4 in Fig. 4. Zero is imputed for missing values, and all variables are scaled. The unit of analysis is a member of Congress-year. Errors are clustered by member. DW-Nominate (partisanship) scores are sourced from Voteview. Fact-checks are from PolitiFact. Facebook posts were processed with Perspective API to generate measures of toxicity. News source quality ratings are from Media Bias Fact Check. News mentions counts mentions of each member of Congress from AP News Wire.

To further investigate correlates of fact-checking, we carry out an exploratory data analysis using LASSO regression to select the most predictive variables or pairwise combinations across all predictors (using 10-fold cross-validation to select the penalty term, lambda, which provides the best balance between bias and variance) (55). Consistent with our previous findings, interactions including a leader term or media mentions are among the most important predictors of the number of fact-checks (Fig. 5).

The interactions of media mentions and being a political leader are associated with the largest increase in the number of fact-checks, 1.5 additional fact-checks per year, the second largest increase in the number of fact-checks per year, is the interaction of being a political leader and having a PolitiFact office in your home state, representing 0.25 additional fact-checks per year. Further, the interaction between members of Congress who are leaders and members of the Republican party is associated with among the largest decreases in the number of fact-checks, while the coefficient for Republicans is nearly zero. After accounting for additional potential relationships among the variables in our study, we again find no evidence that Republicans are fact-checked at a higher rate than Democrats, but do see consistent evidence that more prominent members of Congress are checked more often.

## Discussion

This work informs long-standing questions about biases in fact-checking. Despite previous suggestions, we do not find that Republican members of Congress are fact-checked at a higher rate than Democratic members of Congress. Instead, fact-checks overwhelmingly focus their attention on a small number of prominent political figures. Overall, it appears members’ prominence, rather than their party affiliation, is associated with increased fact-checking. These findings are consistent across several model specifications, functional forms, and transformations of the outcome variable.

Our work has several implications for understanding biases in fact-checking. First, past work has often selected on the dependent variable by only assessing members of Congress that received fact-checks. However, to make valid inferences about potential biases, analyses must include members of Congress who were not fact-checked. Second, researchers should explicitly account for other factors that might influence the rate of fact-checking, rather than implicitly assuming that party identification explains any differences.

Our work also speaks to issues for research which builds on fact-checks. Those using data derived from fact-checking organizations should be mindful that these data focus on a small number of prominent members and do not amount to evaluations of claims by a representative sample of members of Congress. If less prominent members systematically make different kinds of claims, then measures derived from fact-checks may not apply (e.g. a claim-evaluation tool built on Politifact data may not be good at evaluating the veracity of the kinds of claims made by less prominent members).

Our work also has implications for improving the quality of fact-checking. While we find no evidence of evidence of political biases in the number of fact-checks, we do find that Politifact heavily focuses on a small number of members of Congress. The lack of a systematic and transparent approach to selecting what to fact-check leaves the system open to claims of bias. One remedy would be to add checks of claims by a random sample of representatives stratified on state and seniority, rather than checking mostly members that already receive considerable media attention. In addition, clearly documenting the statements that checkers evaluated and the criteria they used to determine if a statement was “fact-checkable” would provide additional clarity to the process. Given the importance of fact-checking to the overall health of the information environment (1–6) and ongoing questions about biases in the process (18–22), it is critical that fact-checking groups are systematic and transparent in their approach.

Some limitations of our study should be noted. First, while Politifact is the largest fact-checking organization (16, 27, 30, 39, 56), and their data are frequently used in academic research (4, 10, 12, 13, 16, 27–30, 39), our findings may not apply to all fact-checking organizations. We do note that others have found a relatively high level of agreement between fact-checking groups (46, 57). Second, while we find no evidence of biases in the number of fact-checks for Republicans and Democrats across numerous specifications, other biases may be present. For instance, regardless of the truthfulness of the underlying statements, they may be rated differently based on the member’s party identification. While the accuracy of fact-checks is outside the scope of this paper, we note that future work aiming to assess whether the correlation between statement truthfulness and rating is different across parties should ensure that they do not select on the dependent variable or assume that party is the only salient factor. Third, building on (41, 58–60), the assessment of potential biases in fact-checking should be further extended to include elected officials outside the United States.

## Materials and methods

### Fact-checking data

Fact-checks used in our analysis are sourced from Politifact, a nonpartisan, nonprofit fact-checking organization which is among the largest and most cited sources for political fact-checks in the United States (25, 30). Politifact was founded by and is run by journalists (26). For each member of Congress, we locate their page on the Politifact site and collect the number of fact-checks for this individual.

### Congressional data

Congressional biographical data including the term dates, party affiliation, chamber, state, and gender are sourced from Congress.gov’s API (61).

### Measures

#### Party and partisanship

We measure partisanship using DW-Nominate score (62, 63). We take the absolute value of this score to capture how far from center a given member leans, regardless of party. In addition, we create an indicator for party affiliation that is coded 1 if a member of Congress is a Republican and 0 otherwise.

#### Political leadership

Political leaders are defined as members of Congress who hold leadership roles in Congress. This includes the Speaker of the House of Representatives, the Senate Minority or Majority Leader, and the majority or minority whip in the House of Representatives. In addition, we include members of Congress who ran for president in the 2016 or 2020 elections. In the Supplementary material, we conduct additional analyses with varying definitions of political leadership and find consistent results.

#### News mentions

Our media prominence measure is a count of mentions of a member of Congress by articles in the Associated Press News Wire during our study period (2018–2021). Each member of Congress’ News Mentions variable is the yearly count of the number of AP News Wire articles where they are mentioned at least once. AP News Wire content was accessed through Nexis Uni’s API. To identify mentions of members of Congress in our search, we consulted the AP Stylebook to select the titles used by AP journalists. From our set of titles and naming conventions, we built a search query that includes all relevant combinations of legislative title and name for a given member-year.

#### Social media presence

Our measures of social media presence are taken from Congressional members’ activity on Facebook within the study dates from 2018 to 2021. These measures include the number of followers for each member and the number of posts shared.

#### Content quality

We generate two measures of the quality of the content shared online by members of Congress. The first measures the toxicity of the content in their Facebook posts. Toxicity scores are generated using Perspective API, a machine learning-based tool developed by Google and Jigsaw, which assigns a score between 0 and 1 to rate the probability of an average Facebook user perceiving a post as toxic. The second is the proportion of domains shared to sites that have been rated as low-quality news sources. We use Media Bias Fact Check, a news quality rating site, to rate the quality of news sites.

#### Structural controls

We refer to Congress members’ tenure and whether Politifact has a state fact-checking office in a member’s home state as structural controls. Tenure is measured as the number of years a member has been in Congress. Members whose home state has a state office are coded 1; other locations are coded 0. Politifact state offices (state editions) are present in California, Florida, Iowa, Michigan, New Hampshire, New York, North Carolina, Pennsylvania, Texas, West Virginia, and Wisconsin.

## Supplementary Material

pgae567_Supplementary_Data

## Data Availability

The data and code necessary to replicate the results in this study are available in the Harvard Dataverse at https://doi.org/10.7910/DVN/D8A7MS.

## Previous Presentation

These results were previously presented at the Stockholm School of Economics, Empirical Studies of Conflict meeting.
