# Supplementary material for: Fact-checks focus on famous politicians, not partisans
Source: PNAS Nexus. 2024 Dec 19;4(1):pgae567. doi: 10.1093/pnasnexus/pgae567 (PMC11732390; doi:10.1093/pnasnexus/pgae567)
Supplement: pgae567_Supplementary_Data [file pgae567_supplementary_data.pdf]

Fact-checks focus on famous politicians, not partisans

Kevin T. Greene,[1*] Nilima Pisharody,[2] Faelynn Carroll,[1] Jacob N. Shapiro[1]

[1]Empirical Studies of Conflict Project, Princeton University
[2]Department of Economics, New York University

*To whom correspondence should be addressed; e-mail: kg2082@princeton.edu.

Materials and Methods

Descriptive Information

This section provides descriptive information for the variables included in our analyses. Table S1 shows the average number of fact-checks per party. Fig. S1 shows the distributions of the variables included in our analyses. Descriptive statistics for the variables included in our main results are included in the main paper as Table 1.

Party	N	Total Fact-Checks	Average Fact-Checks
Democrat	1093	393	0.36
Republican	1077	430	0.399

Table S1: Fact-Checks by Party. The unit of observation is the member of Congress-year. Fact-checks are sourced from PolitiFact for all members of Congress from 2018-2021.

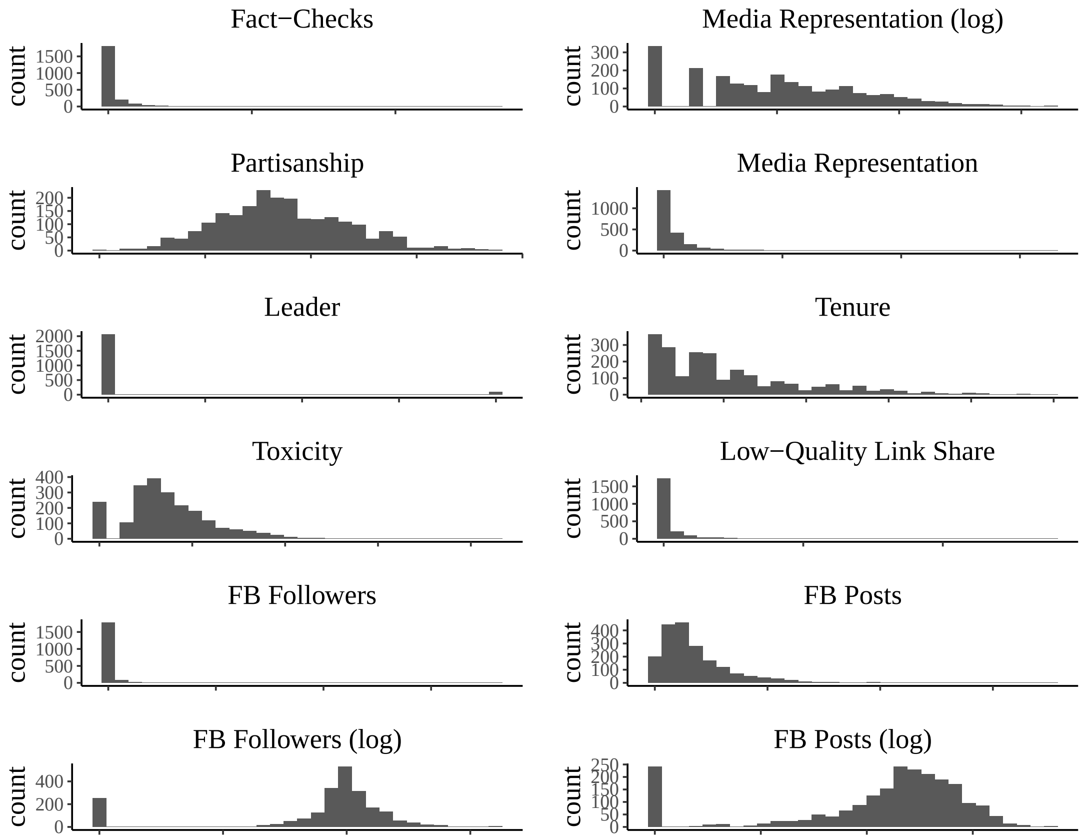

Fig. S1: Distribution of analysis variables. The unit of observation is a member of Congress-year. Data span 2018-2021. Fact-checks are sourced from PolitiFact; partisanship scores are the absolute value of DW Nominate scores from Voteview; Facebook posts were processed through Perspective API to generate measures of toxicity; news links shared within Facebook posts were assigned quality ratings using Media Bias Fact Check, which we take the proportion of to create the "Low-Quality Link Share" variable. Media Prominence is the count of mentions of a member of congress by AP News Wire articles during the study period. We use the logged version of Facebook followers, Facebook posts, and Media Prominence in our main analysis.

Claim Source Origins

Each claim fact-checked by PolitiFact contains a description of the source material where the claim was identified. For instance, the fact check might note the claim was "stated on September 20, 2024 in a 2024 campaign ad." We collect this information for each fact check in our dataset and divide them into five high-level source categories (traditional media, social media,

campaign materials, debates, and Congressional floor).

Traditional media includes interviews or appearances in newspapers, radio or televised media, town halls sponsored by media companies, op-eds, and press conferences. Social media includes posts on Twitter, Facebook, and Instagram. Campaign materials include policy proposals, campaign ads, campaign stops, public forums not hosted by media companies, and other campaign items such as websites, newsletters, and videos. Debates include both televised and non-televised debates. Congressional floor includes speeches made on the floor of the House or Senate and remarks made during Congressional hearings and committee meetings.

Claims made in traditional media represent the single largest source for PolitiFact fact-checking. Social media is also a major source for fact-checked claims, representing roughly 27% of the total. Similarly, roughly 27% of the claimschecked by PolitiFact come directly from candidates' campaign materials.

Source	% Total
Traditional media	33.2
Social media	27.3
Campaign materials	26.7
Debates	7.45
Congressional floor	5.40

Table S2: Percentage of total PolitiFact fact checks of Congressional members (2018-2021) by claim source type.

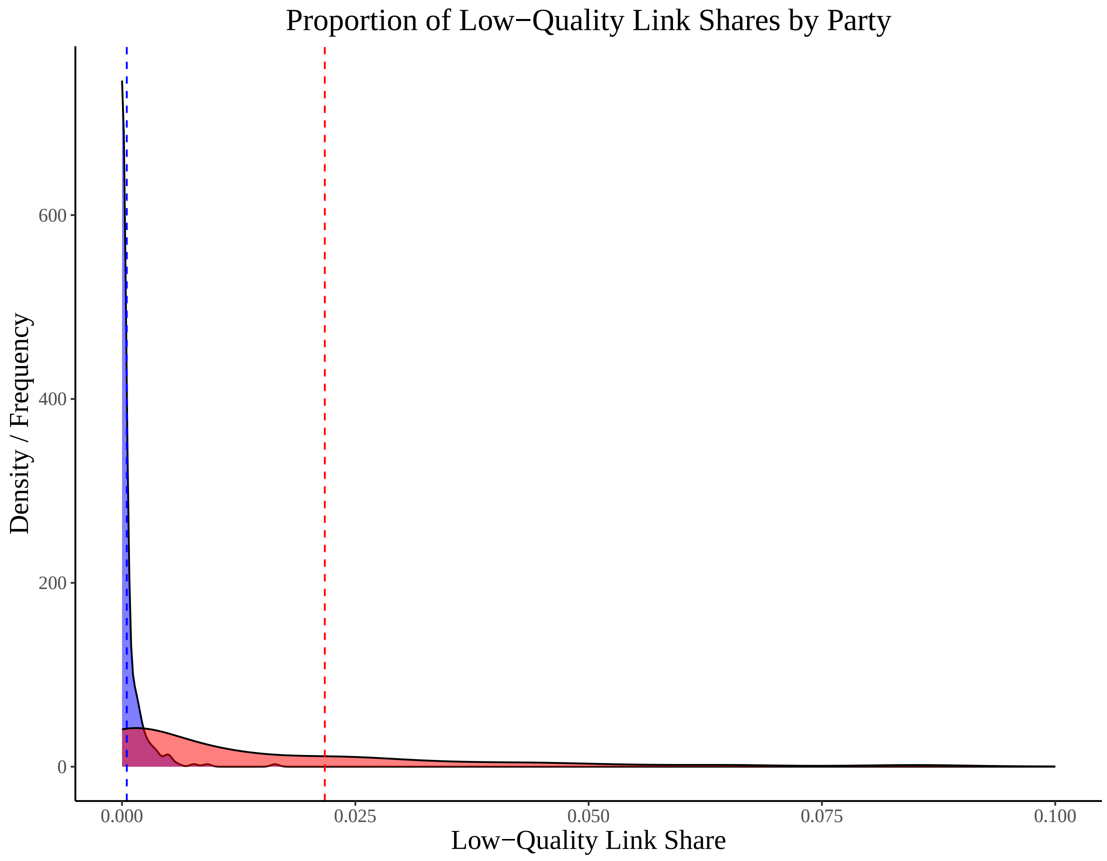

Fig. S2: Density of Low-Quality Link Share by Party. The red shaded region represents the distribution of low-quality link sharing by Republican members of Congress, and the blue shaded region represents Democrats. The mean for each group is represented by a dashed line in each respective color. The fundamentally different shape of each party's low-quality link share distribution motivates using this variable as a control.

Regression Results

Main Results

This section contains the estimating equation and full regression table for the results in the main paper (Fig. 4).

$$
\begin{aligned}
FactChecks_{it} = {} & \beta_1 Party + \beta_2 Partisanship + \beta_3 Tenure + \beta_4 FBFollowers(log) \\
& + \beta_5 PoliticalLeaders + \beta_6 LowQualityLinks + \beta_7 Toxicity \\
& + \alpha_i + \gamma_t + \epsilon_i
\end{aligned} \tag{1}
$$

Equation (1) Is the specification for our main analysis.

	(1)	(2)	(3)	(4)	(5)
**Partisanship**					
Republican	0.040	0.033	0.029	0.028	0.032
	(0.087)	(0.065)	(0.064)	(0.065)	(0.066)
Partisanship		0.067	0.058	0.056	0.048
		(0.046)	(0.041)	(0.041)	(0.038)
**Media Prominence**					
News Mentions (log)		0.352***	0.348***	0.347***	0.353***
		(0.057)	(0.046)	(0.046)	(0.047)
**Political Leadership**					
Leader		1.945***	2.034***	2.033***	1.843***
		(0.467)	(0.379)	(0.378)	(0.337)
**Social Media Presence**					
Followers (log)		0.083*	0.036	0.037	0.011
		(0.033)	(0.029)	(0.030)	(0.069)
Facebook Posts (log)					−0.002
					(0.062)
Facebook Posts (log)*Leader					0.670
					(0.494)
**Content Quality**					
Low-Quality Link Share		0.040	0.046	0.052	0.052
		(0.042)	(0.037)	(0.036)	(0.035)
Toxicity		−0.028	0.003	0.005	0.006
		(0.046)	(0.040)	(0.040)	(0.037)
**Structural Controls**					
Tenure		−0.149***	−0.182***	−0.180***	−0.177***
		(0.029)	(0.041)	(0.040)	(0.037)
State Fact-Check		0.445***			
		(0.076)			
Num.Obs.	2170	2170	2170	2170	2170
R2	0.000	0.232	0.327	0.329	0.338
Mean	0.379	0.379	0.379	0.379	0.379
Std. Dev.	1.393	1.393	1.393	1.393	1.393
State FE			✓	✓	✓
Year FE				✓	✓

* $p < 0.05$, ** $p < 0.01$, *** $p < 0.001$

Table S3: OLS Fixed Effects. Model variables (RHS) are scaled for comparability and missing values are imputed as zero. The unit of observation is a member of Congress-year. Errors are clustered by member. DW-Nominate (partisanship) scores are sourced from Voteview. Fact-checks are from PolitiFact. Facebook posts were processed with Perspective API to generate measures of toxicity; News source quality ratings are from Media Bias Fact Check. News mentions counts mentions of each member of Congress from AP News Wire.

Main Results, Robustness to Scaling and Imputation

In Table S4 we present the full regression table for the results in the main paper (Fig. 4) without rescaling the variables or imputing missing values.

	(1)	(2)	(3)	(4)	(5)
**Partisanship**					
Republican	0.040	0.067	0.081	0.078	0.091
	(0.087)	(0.070)	(0.071)	(0.072)	(0.073)
Partisanship		0.352	0.297	0.293	0.249
		(0.323)	(0.294)	(0.295)	(0.281)
**Media Prominence**					
News Mentions (log)		0.231***	0.224***	0.223***	0.231***
		(0.035)	(0.032)	(0.031)	(0.032)
**Political Leadership**					
Leader		1.794***	1.877***	1.877***	−2.032
		(0.431)	(0.362)	(0.361)	(2.175)
**Social Media Presence**					
FB Followers (log)		0.148**	0.128***	0.129***	0.099***
		(0.055)	(0.036)	(0.036)	(0.030)
FB Posts (log)					0.012
					(0.038)
Posts (log)*Leader					0.770
					(0.461)
**Content Quality**					
Low-Quality Link Share		0.790	0.923	1.108	1.096
		(1.187)	(1.064)	(1.020)	(1.033)
Toxicity		−1.448	0.021	0.046	0.200
		(1.939)	(1.761)	(1.752)	(1.646)
**Structural Controls**					
Tenure		−0.022***	−0.024***	−0.024***	−0.022***
		(0.004)	(0.006)	(0.006)	(0.005)
State Fact-Check		0.473***			
		(0.081)			
Num.Obs.	2170	1912	1912	1912	1912
R2	0.000	0.246	0.339	0.340	0.357
Mean	0.379	0.403	0.403	0.403	0.403
Std. Dev.	1.393	1.451	1.451	1.451	1.451
State FE			✓	✓	✓
Year FE				✓	✓

* $p < 0.05$, ** $p < 0.01$, *** $p < 0.001$

Table S4: OLS Fixed Effects, Scaling and NA Sensitivity. This table reports model estimations without imputing zero for missing values or scaling variables, as done in the data used in the main analysis. The unit of observation is a member of Congress-year. Errors are clustered by member. DW-Nominate (partisanship) scores are sourced from Voteview. Fact-checks are from PolitiFact. Facebook posts were processed with Perspective API to generate measures of toxicity; News source quality ratings are from Media Bias Fact Check. News mentions counts mentions of each member of Congress from AP News Wire.

In Table S5 we present results from M4 in the main paper (Fig. 4), with missing values dropped in Column 1, and the mean imputed for missing values in Column 2.

	(1)	(2)
**Partisanship**		
Republican	0.078	0.018
	(0.072)	(0.064)
Partisanship	0.045	0.053
	(0.045)	(0.040)
**Media Prominence**		
News Mentions (log)	0.314***	0.285***
	(0.044)	(0.041)
**Political Leadership**		
Leader	1.877***	1.870***
	(0.361)	(0.343)
**Social Media Presence**		
FB Followers (log)	0.166***	0.169***
	(0.047)	(0.045)
**Content Quality**		
Low-Quality Link Share	0.042	0.047
	(0.039)	(0.036)
Toxicity	0.001	−0.007
**Structural Controls**		
	(0.037)	(0.034)
Tenure	−0.216***	−0.208***
	(0.051)	(0.044)
Num.Obs.	1912	2170
R2	0.340	0.337
Mean	0.403	0.379
Std. Dev.	1.451	1.393
State FE	✓	✓
Year FE	✓	✓

* $p < 0.05$, ** $p < 0.01$, *** $p < 0.001$

Table S5: OLS Fixed Effects, NA sensitivity. Model variables (RHS) have been scaled for comparability. Both columns use OLS FE M4 from main Table 4. Column 1 drops NAs and Column 2 imputes the mean for NAs. The unit of observation is a member of Congress-year. Errors are clustered by member. DW-Nominate (partisanship) scores are sourced from Voteview. Fact-checks are from PolitiFact. Facebook posts were processed with Perspective API to generate measures of toxicity; News source quality ratings are from Media Bias Fact Check. News mentions counts mentions of each member of Congress from AP News Wire.

Main Results, Robustness to Definition of Political Leadership

In Table S6 we present the full regression table for the results in the main paper (Fig. 4) when measuring political leaders as: the Speaker of the House of Representatives, and the Senate Minority or Majority Leader. Compared to the definition of political leaders used in the main results, this less inclusive definition omits Majority and Minority Whips in the House of Representatives.

	(1)	(2)	(3)	(4)	(5)
**Partisanship**					
Republican	0.040	0.078	0.059	0.058	0.049
	(0.087)	(0.061)	(0.067)	(0.068)	(0.065)
Partisanship		0.050	0.039	0.038	0.037
		(0.045)	(0.041)	(0.041)	(0.038)
**Media Prominence**					
News Mentions (log)		0.339***	0.339***	0.338***	0.336***
		(0.053)	(0.044)	(0.044)	(0.045)
**Political Leadership**					
Leader		2.418***	2.397***	2.397***	1.254*
		(0.623)	(0.500)	(0.499)	(0.580)
**Social Media Presence**					
FB Followers (log)		0.081*	0.044	0.044	0.022
		(0.033)	(0.030)	(0.030)	(0.067)
FB Posts (log)					−0.012
					(0.061)
Posts (log)*Leader					0.604
					(0.464)
**Content Quality**					
Low-Quality Link Share		0.045	0.053	0.060	0.056
		(0.047)	(0.045)	(0.044)	(0.038)
Toxicity		−0.007	0.021	0.023	0.018
		(0.048)	(0.042)	(0.042)	(0.038)
**Structural Controls**					
Tenure		−0.120***	−0.146***	−0.144***	−0.162***
		(0.025)	(0.036)	(0.035)	(0.034)
State Fact-Check		0.441***			
		(0.074)			
Num.Obs.	2170	2170	2170	2170	2170
R2	0.000	0.243	0.330	0.331	0.344
Mean	0.379	0.379	0.379	0.379	0.379
Std. Dev.	1.393	1.393	1.393	1.393	1.393
State FE			✓	✓	✓
Year FE				✓	✓

* $p < 0.05$, ** $p < 0.01$, *** $p < 0.001$

Table S6: OLS Fixed Effects, Leader Definition Sensitivity. Model variables (RHS) have been scaled for comparability, and missing values are imputed as zero. The unit of observation is a member of Congress-year. Errors are clustered by member. DW-Nominate (partisanship) scores are sourced from Voteview. Fact-checks are from PolitiFact. Facebook posts were processed with Perspective API to generate measures of toxicity; News source quality ratings are from Media Bias Fact Check. News mentions counts mentions of each member of Congress from AP News Wire.

Main Results, Robustness to Gender

In this section, we examine the robustness of our results to gender by looking at the share of men and women in the data and how fact-checks are allocated between genders. First, we note the significant imbalance between the number of women in our data, just 177 or about 24.8% of the sample, compared to the number of men, 539 or about 75.2 %. Table S7 displays the 15 most fact-checked politicians within our data. Table S9 and Table S8 display the 15 most fact-checked politicians by gender. Including party affiliation in these tables reveals that Democrats are over represented in all three sets of most fact-checked politicians.

Finally, we add a control for gender to our specifications in Table S10. We find that gender has not statistically significant association with fact-checking in our models and the coefficient is significantly reduced once covariates and controls are added. This suggests that gender is not a significant driver of a Congress members' likelihood of being fact-checked. However, this cannot speak to potential gender biases in who becomes a member of Congress or which members occupy the categories that our models find influence fact-checking rates.

Member of Congress	Total Fact-Checks	Party
Bernie Sanders	53	Democrat
Ted Cruz	27	Republican
Elizabeth Warren	27	Democrat
Tammy Baldwin	22	Democrat
Kamala Harris	22	Democrat
Joe Manchin	22	Democrat
Ron Johnson	21	Republican
Rick Scott	18	Republican
Amy Klobuchar	16	Democrat
Kevin McCarthy	16	Republican
Nancy Pelosi	16	Democrat
Cory Booker	12	Democrat
Kirsten Gillibrand	12	Democrat
Glenn Grothman	12	Republican
Mark Pocan	11	Democrat

Table S7: 15 Most Fact-Checked Politicians, 2018-2021. Fact-check data are from PolitiFact.

Member of Congress	Total Fact-Checks (2018-2021)	Party
Bernard Sanders	53	Democrat
Ted Cruz	27	Republican
Joe Manchin	22	Democrat
Ron Johnson	21	Republican
Rick Scott	18	Republican
Kevin McCarthy	16	Republican
Cory Booker	12	Democrat
Glenn Grothman	12	Republican
Mark Pocan	11	Democrat
Marco Rubio	11	Republican
Ron DeSantis	10	Republican
Beto O'Rourke	9	Democrat
John Cornyn	7	Republican
Tom Cotton	7	Republican
Chris Jacobs	7	Republican

Table S8: 15 Most Fact-Checked Male Politicians, 2018-2021. Fact-check data are from PolitiFact.

Member of Congress	Total Fact-Checks	Party
Elizabeth Warren	27	Democrat
Tammy Baldwin	22	Democrat
Kamala Harris	22	Democrat
Amy Klobuchar	16	Democrat
Nancy Pelosi	16	Democrat
Kirsten Gillibrand	12	Democrat
Gwen Moore	10	Democrat
Martha McSally	9	Republican
Shelley Capito	7	Republican
Tulsi Gabbard	7	Democrat
Vicky Hartzler	6	Republican
Claire McCaskill	6	Democrat
Ilhan Omar	6	Democrat
Veronica Escobar	5	Democrat
Kelly Loeffler	5	Republican

Table S9: 15 Most Fact-Checked Female Politicians, 2018-2021. Fact-check data are from PolitiFact.

	(1)	(2)	(3)	(4)	(5)
**Partisanship**					
Republican	0.084	0.053	0.051	0.051	0.058
	(0.090)	(0.066)	(0.065)	(0.066)	(0.067)
Partisanship		0.067	0.056	0.054	0.045
		(0.046)	(0.040)	(0.040)	(0.038)
**Gender**					
Female	0.165	0.077	0.080	0.085	0.098
	(0.113)	(0.090)	(0.066)	(0.066)	(0.065)
**Media Prominence**					
News Mentions (log)		0.349***	0.344***	0.343***	0.349***
		(0.058)	(0.046)	(0.046)	(0.047)
**Political Leadership**					
Leader		1.954***	2.039***	2.037***	1.847***
		(0.462)	(0.377)	(0.376)	(0.334)
**Social Media Presence**					
FB Followers (log)		0.082*	0.037	0.038	0.015
		(0.033)	(0.029)	(0.030)	(0.069)
FB Posts (log)					−0.006
					(0.062)
**Content Quality**					
Posts (log)*Leader					0.678
					(0.491)
Low-Quality Link Share		0.041	0.047	0.054	0.055
		(0.042)	(0.037)	(0.035)	(0.035)
Toxicity		−0.030	0.001	0.002	0.003
		(0.047)	(0.040)	(0.040)	(0.038)
**Structural Controls**					
Tenure		−0.146***	−0.179***	−0.177***	−0.174***
		(0.030)	(0.041)	(0.041)	(0.037)
State Fact-Check		0.443***			
		(0.077)			
Num.Obs.	2170	2170	2170	2170	2170
R2	0.003	0.232	0.328	0.329	0.338
Mean	0.379	0.379	0.379	0.379	0.379
Std. Dev.	1.393	1.393	1.393	1.393	1.393
State FE			✓	✓	✓
Year FE				✓	✓

* $p < 0.05$, ** $p < 0.01$, *** $p < 0.001$

Table S10: OLS Fixed Effects, Gender Sensitivity. This table reproduces the main results and add gender as a control to check the results' sensitivity. Model variables (RHS) have been scaled for comparability, and missing values are imputed as zero. The unit of observation is a member of Congress-year. Errors are clustered by member. DW-Nominate (partisanship) scores are sourced from Voteview. Fact-checks are from PolitiFact. Facebook posts were processed with Perspective API to generate measures of toxicity; News source quality ratings are from Media Bias Fact Check. News mentions counts mentions of each member of Congress from AP News Wire.

Main Results, Robustness to Outliers

In this section, we transform our outcome variable to account for the skew in the number of fact-checks received by members of Congress. In Table S11 we log transform the variable, while in Table S12 we Winsorize the variable. In some of the models we do see a positive an significant coefficient for Republicans, however, the variable is no longer significant at conventional levels after including either year or state fixed-effects.

	(1)	(2)	(3)	(4)	(5)
**Partisanship**					
Republican	0.057*	0.056*	0.041	0.041	0.042
	(0.027)	(0.023)	(0.023)	(0.023)	(0.023)
Partisanship		0.011	0.007	0.007	0.007
		(0.014)	(0.012)	(0.012)	(0.012)
**Media Prominence**					
News Mentions (log)		0.123***	0.124***	0.124***	0.125***
		(0.015)	(0.013)	(0.012)	(0.012)
**Political Leadership**					
Leader		0.590***	0.640***	0.640***	0.623***
		(0.094)	(0.083)	(0.083)	(0.085)
**Social Media Presence**					
FB Followers (log)		0.030**	0.015	0.015	0.007
		(0.011)	(0.010)	(0.010)	(0.019)
FB Posts (log)					0.007
					(0.019)
FB Posts (log)*Leader					0.059
					(0.095)
**Content Quality**					
Low-Quality Link Share		0.027*	0.028**	0.030**	0.030**
		(0.011)	(0.011)	(0.011)	(0.011)
Toxicity		−0.017	−0.003	−0.002	−0.002
		(0.012)	(0.011)	(0.011)	(0.010)
**Structural Controls**					
Tenure		−0.052***	−0.060***	−0.060***	−0.058***
		(0.009)	(0.010)	(0.010)	(0.010)
State Fact-Check		0.182***			
		(0.022)			
Num.Obs.	2170	2170	2170	2170	2170
R2	0.004	0.273	0.388	0.389	0.390
Mean	0.171	0.171	0.171	0.171	0.171
Std. Dev.	0.431	0.431	0.431	0.431	0.431
State FE			✓	✓	✓
Year FE				✓	✓

* $p < 0.05$, ** $p < 0.01$, *** $p < 0.001$

Table S11: OLS Fixed Effects, Outlier Sensitivity I: Log Fact-Checks. This table re-estimates the main specifications using a logged version of the outcome variable, total fact-checks. Model variables (RHS) have been scaled for comparability. Missing values are imputed as zero. The unit of observation is a member of Congress-year. Errors are clustered by member. DW-Nominate (partisanship) scores are sourced from Voteview. Fact-checks are from PolitiFact. Facebook posts were processed with Perspective API to generate measures of toxicity; News source quality ratings are from Media Bias Fact Check. News mentions counts mentions of each member of Congress from AP News Wire.

	(1)	(2)	(3)	(4)	(5)
**Partisanship**					
Republican	0.102	0.093*	0.059	0.058	0.058
	(0.054)	(0.046)	(0.046)	(0.046)	(0.046)
Partisanship		0.019	0.013	0.012	0.010
		(0.027)	(0.023)	(0.023)	(0.023)
**Media Prominence**					
News Mentions (log)		0.241***	0.244***	0.243***	0.244***
		(0.031)	(0.026)	(0.026)	(0.025)
**Political Leadership**					
Leader		1.121***	1.228***	1.228***	1.194***
		(0.187)	(0.174)	(0.174)	(0.176)
**Social Media Presence**					
FB Followers (log)		0.066**	0.037	0.037	0.035
		(0.023)	(0.019)	(0.020)	(0.041)
FB Posts (log)					−0.004
					(0.041)
Posts (log)*Leader					0.119
					(0.188)
**Content Quality**					
Low-Quality Link Share		0.062*	0.063*	0.067*	0.066*
		(0.027)	(0.026)	(0.026)	(0.026)
Toxicity		−0.046	−0.019	−0.017	−0.016
		(0.025)	(0.021)	(0.021)	(0.021)
**Structural Controls**					
Tenure		−0.102***	−0.113***	−0.113***	−0.113***
		(0.018)	(0.020)	(0.020)	(0.022)
State Fact-Check		0.346***			
		(0.045)			
Num.Obs.	2170	2170	2170	2170	2170
R2	0.004	0.257	0.379	0.380	0.381
Mean	0.313	0.313	0.313	0.313	0.313
Std. Dev.	0.856	0.856	0.856	0.856	0.856
State FE			✓	✓	✓
Year FE				✓	✓

* $p < 0.05$, ** $p < 0.01$, *** $p < 0.001$

Table S12: OLS Fixed Effects, Outlier Sensitivity II: Winsorized Fact-Checks. The outcome variable, total fact-checks, has been winsorized using a threshold of +/- 3 s.d.. Model variables (RHS) have been scaled for comparability, and missing values are imputed as zero. The unit of observation is a member of Congress-year. Errors are clustered by member. DW-Nominate (partisanship) scores are sourced from Voteview. Fact-checks are from PolitiFact. Facebook posts were processed with Perspective API to generate measures of toxicity; News source quality ratings are from Media Bias Fact Check. News mentions counts mentions of each member of Congress from AP News Wire.

Main Results, Robustness to Model Functional Form

In Table S13 we re-estimate the results the main paper (Fig. 4) using Poisson regression.

	(1)	(2)	(3)	(4)	(5)
**Partisanship**					
Republican	0.105	0.302	0.285	0.290	0.304
	(0.234)	(0.186)	(0.177)	(0.177)	(0.173)
Partisanship		−0.031	−0.044	−0.047	−0.038
		(0.085)	(0.074)	(0.074)	(0.075)
**Media Prominence**					
News Mentions (log)		0.883***	0.805***	0.798***	0.811***
		(0.095)	(0.079)	(0.078)	(0.078)
**Political Leadership**					
Leader		1.014***	1.431***	1.421***	1.420***
		(0.231)	(0.233)	(0.233)	(0.231)
**Social Media Presence**					
FB Followers (log)		0.128	−0.016	−0.012	−0.053
		(0.104)	(0.082)	(0.081)	(0.124)
FB Posts (log)					0.075
					(0.134)
Posts (log)*Leader					−0.056
					(0.150)
**Content Quality**					
Low-Quality Link Share		0.034	0.041	0.050	0.052
		(0.035)	(0.039)	(0.038)	(0.039)
Toxicity		0.009	0.156*	0.157*	0.150*
		(0.078)	(0.070)	(0.069)	(0.071)
**Structural Controls**					
Tenure		−0.412***	−0.432***	−0.423***	−0.410**
		(0.094)	(0.123)	(0.122)	(0.127)
State Fact-Check		1.085***			
		(0.150)			
Num.Obs.	2170	2170	2038	2038	2038
R2	0.001	0.358	0.436	0.438	0.438
Mean	0.404	0.404	0.404	0.404	0.404
Std. Dev.	1.434	1.434	1.434	1.434	1.434
State FE			✓	✓	✓
Year FE				✓	✓

* $p < 0.05$, ** $p < 0.01$, *** $p < 0.001$

Table S13: Poisson Fixed Effects. The unit of observation is a member of Congress-year. Errors are clustered by member. DW-Nominate (partisanship) scores are sourced from Voteview. Fact-checks are from PolitiFact. Facebook posts were processed with Perspective API to generate measures of toxicity; News source quality ratings are from Media Bias Fact Check. News mentions counts mentions of each member of Congress from AP News Wire.

Main Results, Accounting for Differences in Types of Content Across Parties

We train BERTopic model (**?**) on the text from all Facebook posts by members of Congress from 2018-2021 to generate 20 representative topics. We examine the share of each content type in the corpus of Facebook posts, and use these representative topics to account for different content types across party.

Topic	Representative Words
COVID Relief	covid19, health, vaccine, coronavirus, pandemic, small, businesses, relief, help, need
Census 2020	census, 2020, 2020census, counted, count, complete, my2020censusgov, accurate, online, 2020censusgov
Climate & Energy	climate, energy, water, communities, clean, national, change, new, act, federal
Education	students, school, education, academy, schools, high, congratulations, college, student, teachers
Family Economics	tax, families, jobs, child, workers, economy, american, americans, democrats, spending
Gun Violence	gun, violence, police, justice, background, lives, act, law, shooting, floyd
Health Care	health, care, coverage, affordable, medicare, cancer, insurance, americans, drug, preexisting
Holidays	day, family, happy, today, honor, police, life, women, community, history
Immigration & Border	border, immigration, president, trump, crisis, security, shutdown, children, immigrants, southern
LGBTQ Rights	lgbtq, equality, total, transgender, discrimination, internet, pride, 24, rights, past
Local Issues	great, food, community, help, work, office, new, farmers, small, today
Mental Health & Addiction	opioid, mental, health, addiction, epidemic, crisis, drug, suicide, substance, help
Middle East	afghanistan, iran, israel, president, allies, war, hate, american, united, biden
Reproductive Rights	abortion, health, life, reproductive, care, unborn, abortions, women, prolife, maternal
Spanish Language	la, el, en, para, que, los, del, puerto, por, rico
Trump & Democracy	president, trump, vote, election, house, impeachment, democracy, voting, people, american
USPS	postal, usps, service, mail, postmaster, dejoy, post, delivery, general, office
Veterans	veterans, service, honor, military, day, va, today, sacrifice, veteran, thank
Violence Against Women	violence, sexual, trafficking, domestic, survivors, women, victims, assault, human, act
Youth Outreach	art, competition, congressional, app, high, school, students, artwork, challenge, winner

Table S14: Most Representative Words per Topic. Topics were generated using BERTopic on Congress members' Facebook posts. Representative words are selected by BERTopic in the topic generation process.

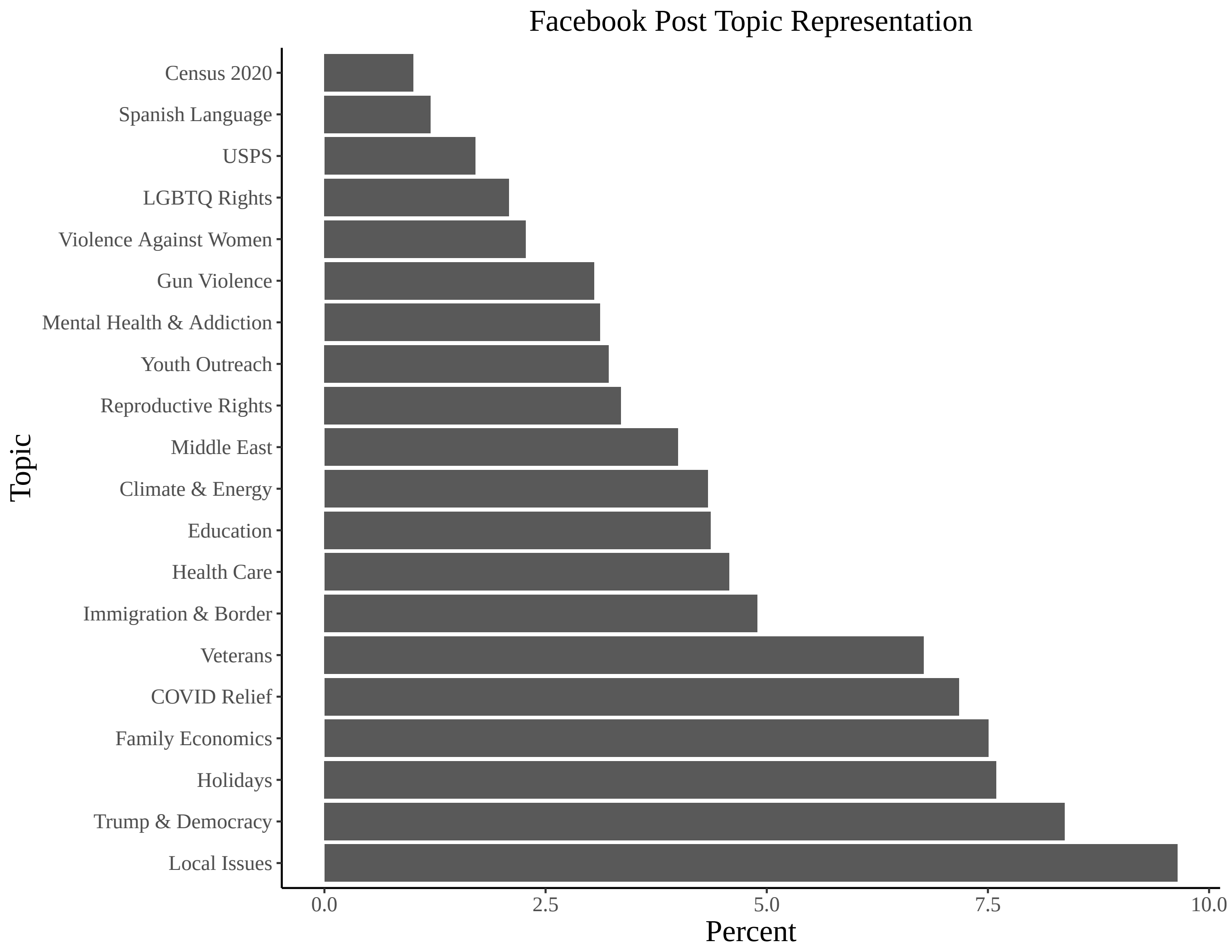

Fig. S3: Distribution of Topics by Percentage of Total Posts. This chart shows how likely a given topic is to be present in the corpus of Facebook posts, out of the set of 20 topics within 865,355 total posts. BERTopic model was used to generate topics from Congress members' Facebook posts.

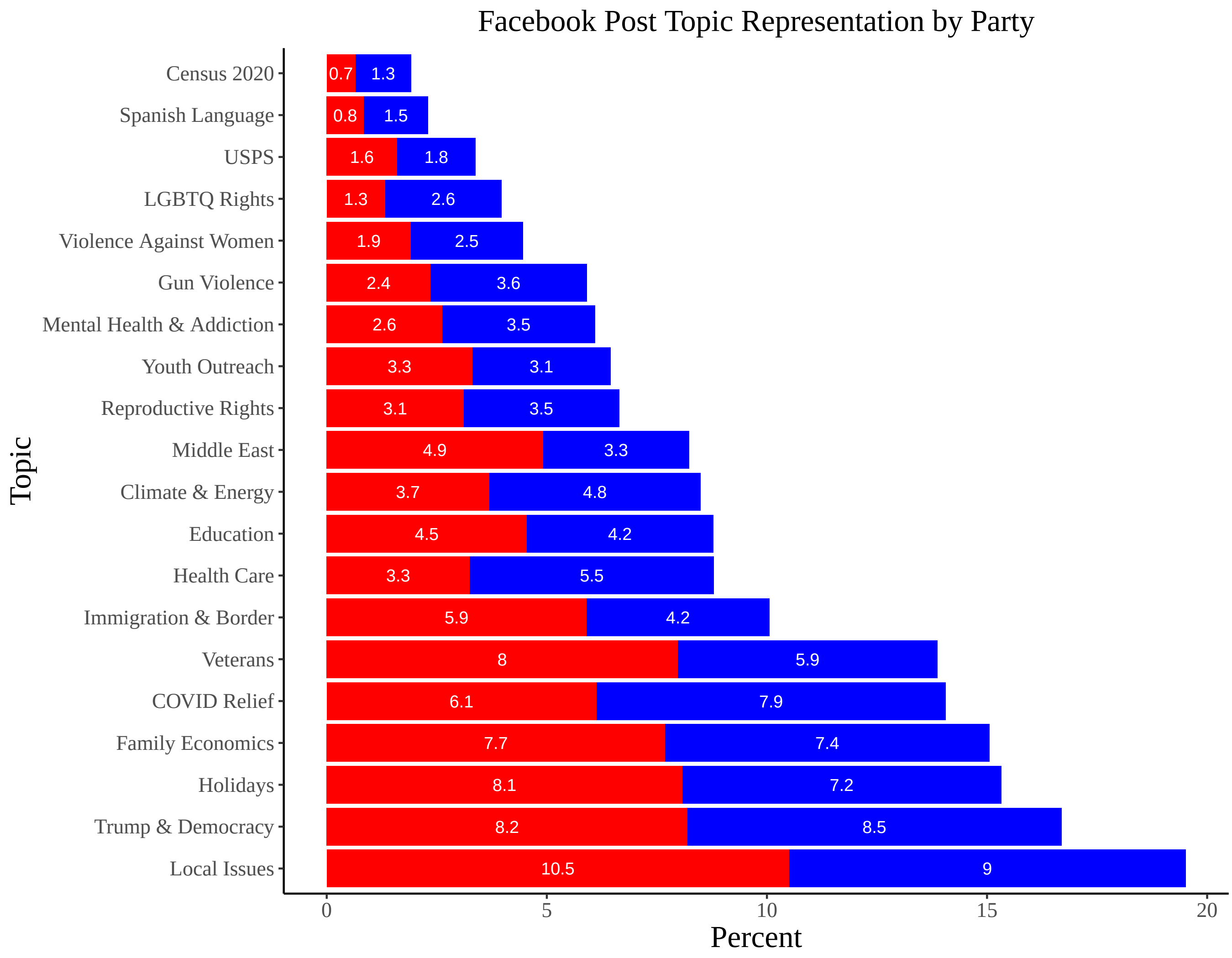

Fig. S4: Distribution of Topics by Percentage of Party's Total Posts. This chart shows how likely a given topic is to be present in the corpus of each party's Facebook posts (either Democrats' or Republicans'), out of the set of 20 topics within 499,219 posts by Democrats and 366,136 posts by Republicans. Facebook posts are sourced from CrowdTangle. BERTopic model was used to generate topics from Congress members' Facebook posts.

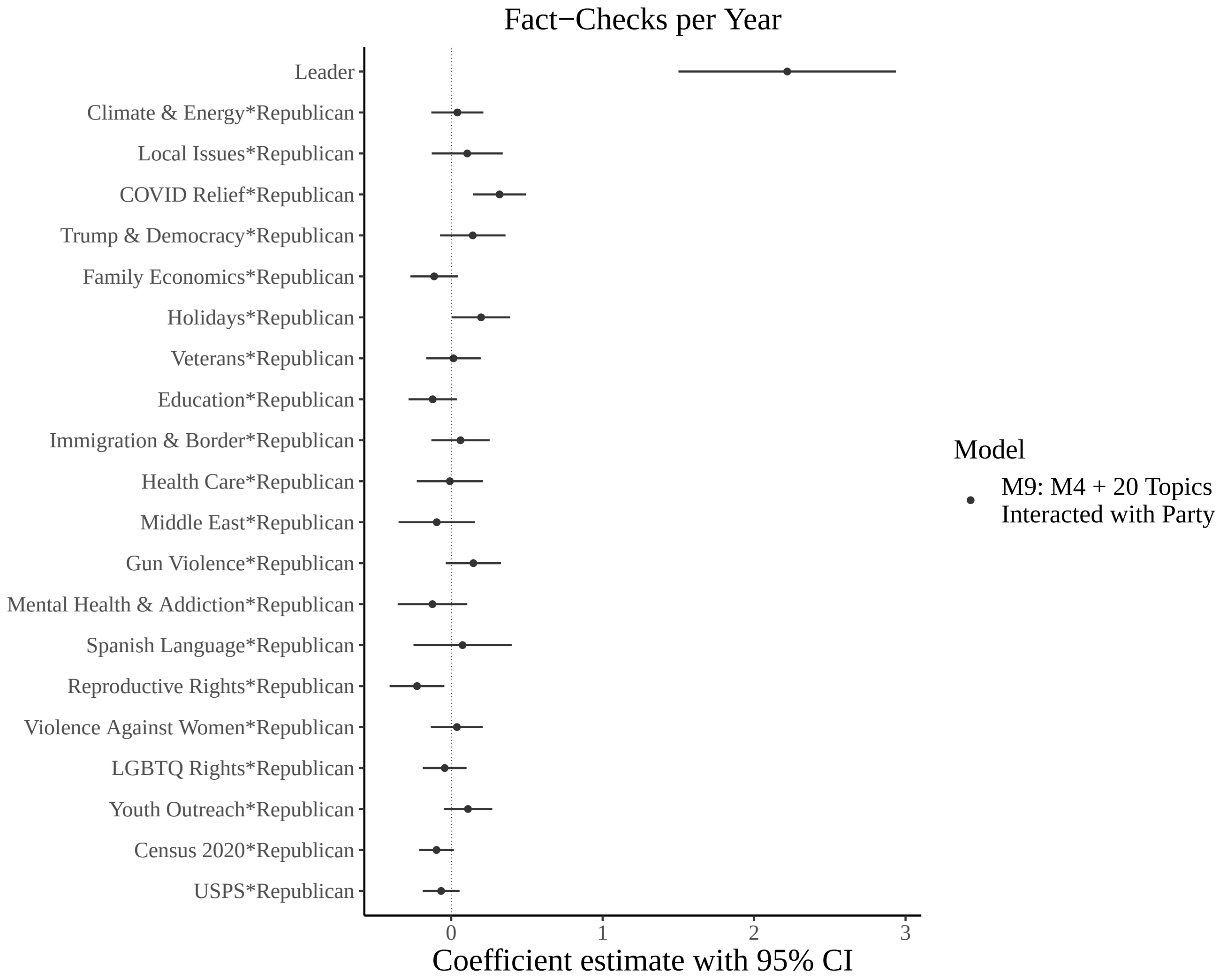

Fig. S5: OLS Fixed Effects Coefficients, 20 Topic Model. BERTopic model was used to generate topics from Congress members' Facebook posts. The constituent terms party, partisanship, leader, tenure, Facebook followers (log), and Facebook posts (log), and post toxicity from our main fixed effects regression model, M4 from Table 4 in the main paper, are included in the model, but omitted here for clarity. The unit of observation is a member of Congress-year. Errors are clustered by member. DW-Nominate (partisanship) scores are sourced from Voteview. Fact-checks are from PolitiFact. Facebook posts were processed with Perspective API to generate measures of toxicity; News source quality ratings are from Media Bias Fact Check.

$$
\begin{aligned}
FactChecks_{it} = {} & \beta_1 Party + \beta_2 Partisanship + \beta_3 Tenure + \beta_4 FBFollowers(log) \\
& + \beta_5 PoliticalLeaders + \beta_6 LowQualityLinks + \beta_7 Toxicity \\
& + \sum_{i=1}^{20} (\delta_i Topic_i + \zeta_i Party \cdot Topic_i) + \alpha_i + \gamma_t + \epsilon_i
\end{aligned} \tag{2}
$$

Equation (2) includes the variables from the main analysis and adds interactions of 20 topics generated using BERTopic modeling of Congress members' Facebook posts.

OLS Fixed Effects, 20 Topic Model

	(1)
**Partisanship**	
Republican	0.012
	(0.083)
Partisanship	0.070
	(0.045)
**Media Prominence**	
News Mentions (log)	0.324***
	(0.043)
**Political Leadership**	
Leader	1.919***
	(0.343)
**Social Media Presence**	
FB Followers (log)	0.154*
	(0.074)
Low-Quality Link Share	0.053
	(0.043)
**Content Quality**	
Toxicity	0.092
	(0.083)
Tenure	−0.181***
	(0.041)
**Structural Controls**	
Local Issues	−0.147
	(0.114)
**Topics**	
COVID Relief	−0.242**
	(0.077)
Trump & Democracy	−0.262*
	(0.126)
Family Economics	0.081
	(0.066)
Holidays	−0.185*
	(0.074)
Veterans	0.015
	(0.072)

Education	0.077
	(0.073)
Immigration & Border	−0.121
	(0.075)
Health Care	0.042
	(0.090)
Middle East	0.127
	(0.103)
Gun Violence	−0.093*
	(0.039)
Mental Health	0.143
	(0.076)
Spanish Language	0.022
	(0.024)
Reproductive Rights	0.120
	(0.077)
Violence Against Women	−0.071
	(0.077)
LGBTQ Rights	0.055
	(0.052)
Youth Outreach	−0.096
	(0.056)
Census 2020	−0.011
	(0.022)
USPS	0.038
	(0.035)
Small Business*Republican	0.142
	(0.118)
**Topics*Republican**	
COVID Relief*Republican	0.317***
	(0.082)
Trump & Democracy*Republican	0.123
	(0.108)
Family Economics*Republican	−0.099
	(0.078)
Holidays*Republican	0.160
	(0.089)
Veterans*Republican	0.023
	(0.087)
Education*Republican	−0.125
	(0.080)

Immigration & Border*Republican	0.083
	(0.093)
Health Care*Republican	−0.047
	(0.111)
Middle East*Republican	−0.104
	(0.126)
Gun Violence*Republican	0.119
	(0.088)
Mental Health & Addiction*Republican	−0.113
	(0.106)
Spanish Language*Republican	0.047
	(0.148)
Reproductive Rights*Republican	−0.174*
	(0.089)
Violence Against Women*Republican	0.029
	(0.087)
LGBTQ Rights*Republican	−0.048
	(0.069)
Youth Outreach*Republican	0.119
	(0.077)
Census 2020*Republican	−0.061
	(0.053)
USPS*Republican	−0.074
	(0.059)
Num.Obs.	2170
R2	0.363
Mean, Fact-Checks Total	0.379
Std. Dev., Fact-Checks Total	1.393
State FE	✓
Year FE	✓

* p < 0.05, ** p < 0.01, *** p < 0.001

Table S15: OLS Fixed Effects, 20 Topic Model. BERTopic model was used to generate topics from Congress members' Facebook posts. Climate and Energy is the omitted topic in the model. The unit of observation is a member of Congress-year. Errors are clustered by member. DW-Nominate (partisanship) scores are sourced from Voteview. Fact-checks are from PolitiFact. Facebook posts were processed with Perspective API to generate measures of toxicity; News source quality ratings are from Media Bias Fact Check. News mentions counts mentions of each member of Congress from AP News Wire.

Omitted Variable Bias Sensitivity

Outcome: *Fact-Checks Per Year*

Treatment:	Est.	S.E.	t-value	$R^2_{Y\sim D\|\mathbf{X}}$	$RV_{q=1}$	$RV_{q=1,\alpha=0.05}$
Party (Republican)	0.028	0.065	0.431	0%	0.9%	0%
Leader	2.033	0.378	5.378	1.3%	10.9%	7.1%
df = 2170						

Table S16: Cinelli Hazlett Omitted Variable Bias Sensitivity. Cinelli and Hazlett's sensitivity test estimates how resilient the treatment variable, party, and the most significant covariate, political leader, are to a possible confounding variable in the OLS regression M4 from our main OLS fixed effects estimations presented in Table S3. M4 includes: party, partisanship, log news mentions, tenure, log Facebook followers, log Facebook posts, the proportion of low quality links shared on Facebook, and political leaders with fixed-effects on state and year, and errors clustered by member of Congress. Note that variables in the regression are scaled and missing values are imputed as zero. Since party is not significant within this model, it is highly sensitive to confounders and could be rendered null by a confounder that explains just 0.9 percent of the DV. In contrast, it would take a confounder that explains 10.9 percent of the DV to rendered leader null due to a confounding variable.

Sensitivity Table S16 displays the results from a Cinelli Hazlett Omitted Variable Bias Sensitivity analysis (*1*) for our treatment variable, party, when it is equal to Republican, and the most significant covariate that we find, congressional leader. Results presented use the point estimates and standard errors from M4 in our main results, presented in Table S3, which is an OLS fixed effects regression of party, partisanship, log news mentions, tenure, log Facebook followers, log Facebook posts, the proportion of low quality links shared on Facebook, and political leaders with fixed-effects on state and year, and errors clustered by member of Congress. Note that variables in the regression are scaled and missing values are imputed as zero. The first column reports the point estimates, the second column reports the standard errors, the third column reports the t-values, the fourth column reports the partial R-squared of the treatment with the outcome, the fifth column reports the robustness value for the point estimate, and the sixth column reports the robustness value for the t-statistic. Since party is not significant, it is highly

sensitive to confounders and the partial r-squared of party with fact-checking is zero (Column 4). If unobserved confounders explain 0.9 percent of the residual variance both of the treatment and the outcome (Column 5) then the estimated effect would be null. Finally, the robustness value for the t-statistic on party (Column 6) means that unobserved confounders that explain any amount of residual variance both of the treatment and of the outcome would mean that the null hypothesis that the true treatment effect is equal to 0 would not be rejected with a significance level of 0.05. Contrastingly, congressional leader, our most significant covariate would require a confounder that explains at least 1.3 percent of the residual variance of the treatment (Column 4) to account for the observed estimated effect, it would take confounders that explain at least 10.9 percent of the residual variance both of the treatment and the outcome to render the estimated effect null, and it would take a confounder that explains at least 7.1 percent of the residual variance on the outcome and the treatment not to reject the null hypothesis that the true treatment effect is zero at a 0.5 significance level.

References

1. Carlos Cinelli and Chad Hazlett. Making sense of sensitivity: Extending omitted variable bias. *Journal of the Royal Statistical Society Series B: Statistical Methodology*, 82(1):39–67, 2020.
